# Combined Anti-Angiogenic Therapy Targeting PDGF and VEGF Receptors Lowers the Interstitial Fluid Pressure in a Murine Experimental Carcinoma

**DOI:** 10.1371/journal.pone.0008149

**Published:** 2009-12-04

**Authors:** Agnieszka Kłosowska-Wardęga, Yoko Hasumi, Mikhail Burmakin, Aive Åhgren, Linda Stuhr, Ingrid Moen, Rolf K. Reed, Kristofer Rubin, Carina Hellberg, Carl-Henrik Heldin

**Affiliations:** 1 Biomedical Center, Ludwig Institute for Cancer Research, Uppsala University, Uppsala, Sweden; 2 Department of Biomedicine, University of Bergen, Jonas Lies vei 91, Bergen, Norway; 3 Biomedical Center, Department of Medical Biochemistry and Microbiology, Uppsala University, Uppsala, Sweden; Karolinska Institutet, Sweden

## Abstract

Elevation of the interstitial fluid pressure (IFP) of carcinoma is an obstacle in treatment of tumors by chemotherapy and correlates with poor drug uptake. Previous studies have shown that treatment with inhibitors of platelet-derived growth factor (PDGF) or vascular endothelial growth factor (VEGF) signaling lowers the IFP of tumors and improve chemotherapy. In this study, we investigated whether the combination of PDGFR and VEGFR inhibitors could further reduce the IFP of KAT-4 human carcinoma tumors. The tumor IFP was measured using the wick-in-needle technique. The combination of STI571 and PTK/ZK gave an additive effect on the lowering of the IFP of KAT-4 tumors, but the timing of the treatment was crucial. The lowering of IFP following combination therapy was accompanied by vascular remodeling and decreased vascular leakiness. The effects of the inhibitors on the therapeutic efficiency of Taxol were investigated. Whereas the anti-PDGF and anti-VEGF treatment did not significantly inhibit tumor growth, the inhibitors enhanced the effect of chemotherapy. Despite having an additive effect in decreasing tumor IFP, the combination therapy did not further enhance the effect of chemotherapy. Simultaneous targeting of VEGFR and PDGFR kinase activity may be a useful strategy to decrease tumor IFP, but the timing of the inhibitors should be carefully determined.

## Introduction

The development of tissue stroma is controlled by several growth factors and cytokines. Platelet-derived growth factor (PDGF) is particularly important for proliferation and chemotaxis of connective tissue cells (reviewed in [1]). PDGF is a family of homo- and hetero-dimeric molecules of structurally related A-, B-, C- and D-polypeptide chains, which exert their cellular effects by binding to α- and β-tyrosine kinase receptors. Vascular endothelial growth factor (VEGF) is the prototype of a five-membered family which control angiogenesis and lymphangiogenesis; the VEGF isoforms also act via tyrosine kinase receptors, *i.e.* the VEGF receptor 1, 2 and 3 [2].

Solid tumors often have an increased interstitial fluid pressure (IFP) which perturbs transcapillary transport and thus is an obstacle in tumor treatment with chemotherapy [3]. The reasons for the increased tumor IFP include leakiness of tumor vessels, *e.g.* as a consequence of overexpression of VEGF [4], which has a well-characterized vascular permeability effect. Administration of the anti-VEGF antibody bevacizumab to patients with colorectal cancer decreased IFP and vessel leakiness [5]. Moreover, overexpression of PDGF can also contribute to increased IFP of tumors, since PDGF inhibition decreases tumor IFP [6]. In normal tissues, PDGF regulates interstitial fluid pressure [7] by acting on stromal fibroblasts and causing an integrin-mediated contraction of the cells that affects the extracellular matrix [8]. Since treatment with either VEGF antagonists [5], [9], [10] or PDGF antagonists [6], [11], [12] have been found to lower tumor IFP, and in view of the potential clinical utility of lowering tumor IFP to enhance chemotherapy, we investigated if the combination of anti-VEGF and anti-PDGF treatment gives a synergistic lowering effect on tumor IFP.

## Results

### Combination of PDGF and VEGF Receptor Kinase Inhibitors Lowers Tumor IFP

KAT-4 tumors were grown subcutaneously in SCID mice. We used the low molecular weight compound imatinib (Glivec, STI571) as a PDGF receptor tyrosine kinase inhibitor at 100 mg/kg body weight. As a VEGF receptor kinase inhibitor, we used PTK/ZK at 25 mg/kg body weight; at this concentration PTK/ZK inhibits the VEGF receptor kinases, but has minimal effects on other kinases such as the PDGF receptor kinases [13].

**Figure 1:**
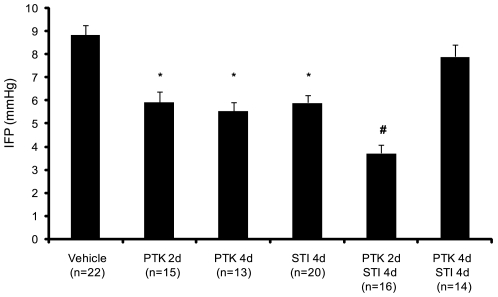
Combination of PDGF and VEGF receptor kinase antagonists lowers tumor IFP. Mice with KAT-4 tumors grown subcutaneously were treated with vehicle, STI571, PTK/ZK, or with combinations of STI571 and PTK/ZK. The IFP of the tumors were measured by the wick-in-needle technique. Data are presented as means +/− SEM. Statistically significant differences (p<0.05) compared to vehicle and long term combination treatment (*), and to all groups (#) are indicated.

Consistent with our previous findings [11], treatment with STI571 for 4 days lowered the IFP of KAT-4 tumors (Fig. 1). Treatment of these tumors with PTK/ZK for 2 or 4 days also lowered the tumor IFP. Moreover, the combination of STI571 treatment for 4 days and PTK/ZK treatment for the last 2 of these days (termed short term combination treatment) gave an additive effect, whereas treatment with both STI571 and PTK/ZK for the full 4 days (termed long term combination treatment) gave a result similar to vehicle treatment (Fig. 1).

### Combination Treatment Affects Tumor Vascularization

**Figure 2:**
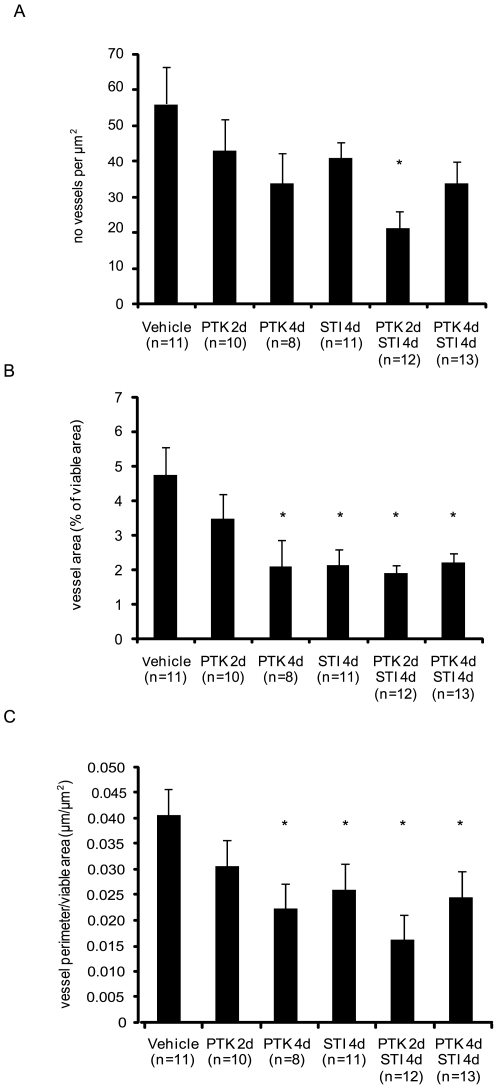
Effects of PDGF and VEGF receptor kinase inhibitors on tumor vascularization. Sections of KAT-4 tumors from mice treated with vehicle, STI571 or PTK/ZK, alone or in combination were stained for endothelial cells of blood vessels by CD31 antibodies and then subjected to stereological analysis. Data are presented as means +/− SEM. Asterisks indicate statistically significant differences (p<0.05) compared to control (vehicle treatment).

To investigate the effect of anti-PDGF and anti-VEGF treatment on tumor vascularization, tumor sections were stained with CD31 antiserum to visualize endothelial cells, followed by stereological analysis. Upon short term, but not long term, combination treatment, the number of vessels decreased (Fig. 2A, Fig. S1). STI571 given for four days decreased the total vessel area and the vessel perimeter, as did the 4 day treatment with PTK/ZK as well as long and short term combination treatment (Fig. 2B,C, Fig. S1). Despite lowering IFP, PTK/ZK given for two days had no effect on the vessel number and morphology (Fig. 2, Fig. S1).

### Anti-Angiogenic Therapy Affects Pericyte Coverage of Vessels

**Figure 3:**
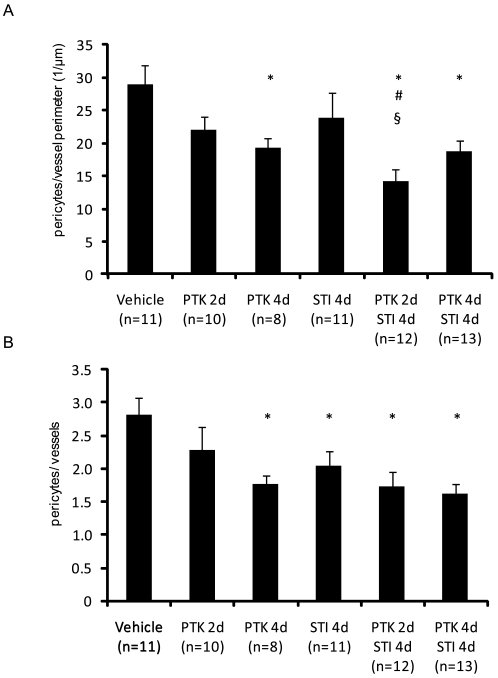
Effect of PDGF and VEGF receptor kinase inhibitors on desmin-positive pericyte coverage. Sections of KAT-4 tumors from mice treated with vehicle, STI571 or PTK/ZK, alone or in combination were stained with antibodies against the pericyte marker desmin. The number of desmin-positive pericytes per vessel perimeter (panel A) or per number of vessels (panel B) were quantified. Data are presented as means +/− SEM. Statistically significant differences (p<0.05) compared to control (vehicle treatment;*), compared to PTK/ZK 2 days (#), and compared to STI571 4 days (§), are indicated.

We also determined the effect of combination treatment on the coverage of vessels by desmin-positive pericytes [14]. Both the number of desmin-positive pericytes per perimeter (Fig. 3A, Fig. S1) and the number of desmin-positive pericytes per vessel (Fig. 3B) were decreased upon treatment with PTK/ZK for 4 days, as well as with both combination treatments. Also, the number of desmin-positive pericytes per vessel was lowered following treatment with STI571 (Fig. 3B, Fig. S1).

### Enhanced Tumor Cell Apoptosis

**Figure 4:**
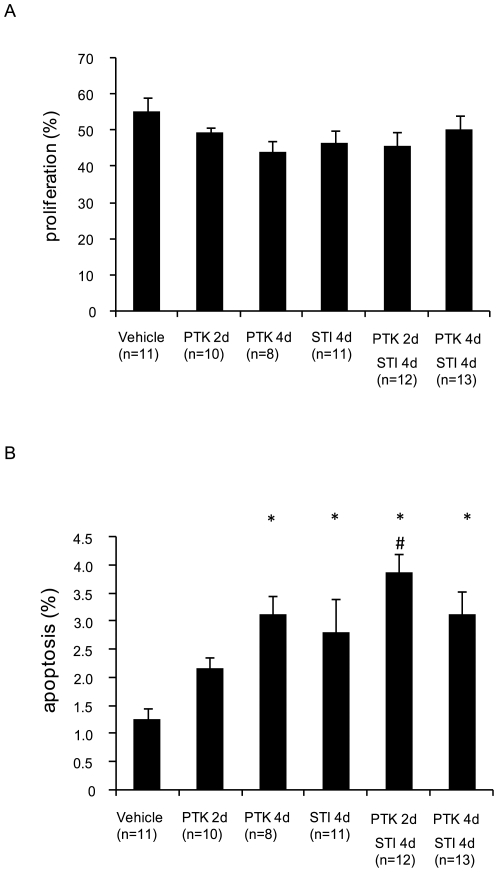
Effect of PDGF and VEGF receptor kinase inhibitors on KAT-4 tumor cell proliferation and apoptosis. Sections of KAT-4 tumors from mice treated with vehicle, STI571 or PTK/ZK, alone or in combinations, were stained for Ki67 (panel A) and cleaved caspase-3 (panel B) to monitor proliferation and apoptosis, respectively. Data are presented as means +/− SEM. Statistically significant differences (p<0.05) compared to control (vehicle treatment;*), and compared to PTK/ZK 2 days (#), are indicated.

To determine the effect of the therapies on tumor cell apoptosis and proliferation, tumor sections were stained with antibodies recognizing cleaved caspase-3 and Ki67, respectively. As seen in Fig. 4A and in Fig. S2, no effect was observed by any of the treatments on tumor cell proliferation. On the other hand, both monotherapies with STI571 or PTK/ZK for 4 days, as well as both short-term and long-term combination therapy enhanced apoptosis of tumor cells (Fig. 4B, Fig. S3).

### Effect of Anti-PDGF and Anti-VEGF Treatment on Vascular Leakage

**Figure 5:**
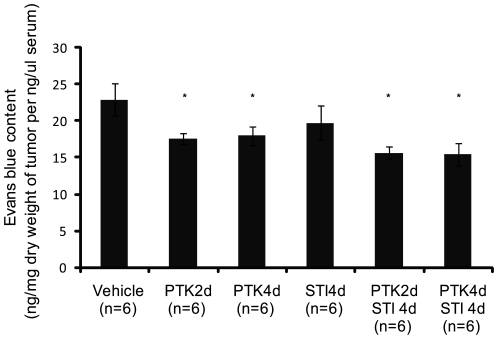
Effect of PDGF and VEGF receptor kinase inhibitors on vessel leakage. Evans blue was injected into the blood stream of tumor bearing mice. The dye in the tumor tissue was extracted and determined spectrophotometrically at 600 nm. The result was presented as the amount of Evans blue [4] extracted from dry weight of the tumor tissue (mg) and divided by the amount of Evans blue [4] present in the 1 µl of serum. Data are presented as means +/− SEM. Statistically significant differences (p<0.05) compared to vehicle treatment (*) are indicated.

The effect of PDGF and VEGF receptor inhibitors on tumor vessel leakiness was determined by injection of Evans blue, a dye that quantitatively binds to albumin in the circulation. The tumor vessel leakage was quantified by dye extraction from excised tumors. Treatment of KAT-4 tumor-bearing mice with VEGF receptor inhibitor alone or in combination with PDGF receptor inhibitor significantly lowered vessel leakiness (Fig. 5). This effect was not observed after treatment by STI571 alone.

### Effect of Anti-PDGF and Anti-VEGF Treatment on Extracellular Volume and Plasma Volume

The extracellular volume (ECV) and plasma volume (PV) of tumor and skin were determined by the dilution of ^51^Cr-EDTA and ^125^I-labeled serum albumin, respectively. The ECV of tumor, as well as skin, was significantly elevated after 4 days of treatment with STI571 (Table 1), whereas treatment with PTK/ZK alone had no significant effect on ECV of tumors or skin. The short term combination treatments had no significant effect on the total tissue water, ECV and PV of the tumor, but it increased both PV and ECV in the skin (Table 1). The long term combination treatment increased the mean tumor ECV, but with large variations within the group, suggesting that vasculature may be undergoing functional remodeling.

**Table 1:** Extracellular volume (ECV), plasma volume (PV) and total tissue water (TTW) measured in the animals treated with either PBS, PTK/ZK, STI571 as well as short and long term combination treatment.

	Tumor			Skin		
	TTW (mL/g dry weight)	PV (mL/g dry weight)	ECV (mL/g dry weight)	TTW (mL/g dry weight)	PV (mL/g dry weight)	ECV (mL/g dry weight)
**PBS (n = 10)**	4.756±0.668	0.041±0.019	1.612±0.451	1.944±0.406	0.008±0.005	1.357±0.430
**STI571 4d (n = 7)**	5.162±0.208	0.047±0.020	**2.187±0.321** *	2.804±1.404	0.017±0.006§	2.053±0.851§
**PTK/ZK 4d (n = 8)**	4.977±0.538	0.052±0.033	1.533±0.311	2.573±0.744	0.018±0.012	1.614±0.422
**PTK/ZK 2d/STI571 4d (n = 7)**	4.658±0.876	0.156±0.250	1.515±0.481	3.122±1.279	**0.110±0.106** *	**2.399±1.097** *
**PTK/ZK 4d/STI571 4d (n = 6)**	5.187±0.789	0.046±0.026	2.140±0.587	2.348±0.672	0.016±0.006	1.704±0.527

All results are presented as mean and S.E.M. Analysis of statistical significance was evaluated with 1-way ANOVA followed by the post-hoc Dunn's test.

* *p*<0.05 compared to PBS treatment.

§ *p*<0.05 compared to PBS treatment when removing the highest outlying data point in PTK/ZK 2d/STI571 4d treatment group.

### Effect of Anti-PDGF and Anti-VEGF Treatment on the Effect of Chemotherapy

**Figure 6:**
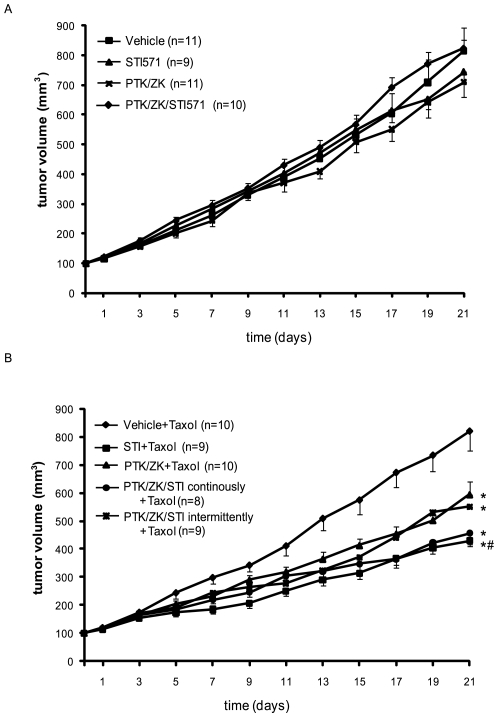
Effect of the combination of chemotherapy, and PDGF and VEGF receptor kinase inhibitors on tumor growth. KAT-4 tumor cells were inoculated subcutaneously in mice which then were treated with daily doses of STI571 or PTK/ZK or both (panel A). B. Taxol (5 mg/kg) was given every five days in combination with daily doses of STI571 or PTK/ZK, alone or in combination (continuous treatment) Alternatively, STI571 was given for four days in combination with PTK/ZK for the last two days prior to Taxol injection (intermittent treatment). Data are presented as means +/− SEM. Statistically significant differences (p<0.05) compared to control (vehicle+Taxol treatment; *) or STI571+Taxol vs PTK/ZK+Taxol (panel B; #), are indicated.

When mice inoculated with KAT-4 tumors were treated with STI571 or PTK/ZK, alone or in combination, no significant effect on tumor growth was observed (Fig. 6A). Likewise, when the animals were treated with Taxol at a concentration of 5 mg/kg, no significant effect on tumor growth was observed. However, when Taxol treatment was combined with STI571 and PTK/ZK, alone or in combination, the tumor growth were reduced (Fig. 6B). The group treated with STI571 together with Taxol had significantly reduced growth rate compared to the group treated with Taxol only, and to the group treated with PTK/ZK together with Taxol. Combination of STI571 and PTK/ZK, either continuously or intermittently for four and two days before the weekly administration of Taxol did not give any enhanced effects compared to monotreatment (Fig. 6B).

## Discussion

Previous studies have shown that anti-PDGF treatment lowers the IFP of tumors [6] and improves the effect of chemotherapy [11]. Since both STI571 and the more specific PDGF aptamers exerted similar effects in KAT-4 tumors on regulation of IFP and Taxol treatment efficiency [11], the observed effects of STI571 are likely to be mediated by inhibition of PDGFR. The effect is presumably mediated by a relaxation of connective tissue cells, which participate in the control of tissue IFP by integrin-mediated contacts with extracellular matrix fibers [8]. It is also possible that anti-PDGF treatment results in a rebuilding of the extracellular matrix of the stroma into a less dense structure allowing a reduction of IFP [15]. However, STI571 inhibits also other kinases such as the stem cell factor receptor, Abl, Arg [16] and DDR1 [17]; therefore, although unlikely, one cannot exclude the possibility that inhibition of these pathways contributed to the lowering of the IFP. In addition, anti-VEGF treatment has also been shown to lower tumor IFP [5], [9], [10]. Since VEGF has a well-characterized vascular permeability effect, it is likely that anti-VEGF lowers tumor IFP by lowering vessel leakiness and thereby lowering the colloid osmotic pressure of the interstitium of tumors.

Thus, anti-PDGF and anti-VEGF treatment most likely affect tumor IFP by different mechanisms. We therefore explored the possibility that the combination of anti-PDGF and anti-VEGF treatment would give an additive effect. Our results indeed show that the combination treatment gives a more efficient lowering of the IFP of the human carcinoma KAT-4 grown subcutaneously in SCID mice. This effect is most likely due to the additive effect of tumor stroma relaxation and decreased plasma protein leakage. In addition, anti-VEGFR therapy has been suggested to normalize the tumor vasculature, thereby temporarily improving their function [18] which might cause improved delivery of the PDGFR inhibitor.

A notable observation was, however, that the timing of the anti-VEGF treatment was of importance when given in combination with anti-PDGF treatment. Thus, two days of treatment efficiently lowered tumor IFP, but not four days of treatment (Fig. 1). The reason for this finding is not clear, but may be related to a transient vessel normalization after anti-VEGF administration followed by a deterioration of the vessels [18]. The decreased functionality of the vessels upon prolonged anti-VEGF treatment could then be accompanied by a decreased delivery of PDGF receptor inhibitor. Although both short and long term combination therapy decreased vessel leakiness (Fig. 5) only short term combination treatment gave a more pronounced anti-angiogenic effect (Fig. 2, Fig. S1) and decreased pericyte coverage (Fig. 3, Fig. S1), compared to long term combination treatment and to the monotherapies.

We have previously shown that treatment with bevacizumab lowers protein leakage as well as ECV in KAT-4 carcinoma [10], whereas in the present study mono-treatment with PTK/ZK had effect only on plasma protein leakage but not ECV. This discrepancy could at least partly depend on the fact that the processes studied are transient, and that there are pharmacokinetic differences between treatment with monoclonal antibodies and low molecular weight inhibitors. In addition, PTK/ZK at high concentrations also targets other kinases including PDGFR [19]. We therefore used a modest concentration of PTK/ZK to avoid inhibition of PDGFR [13]. It is possible that those modest concentrations of PTK/ZK did not correct vessel leakiness equally efficient as treatment with bevacizumab.

Treatment of experimental carcinoma with prostaglandin E_1_ transiently lowers IFP, increase transport of low molecular weight tracers and of 5-fluorouracil through the tumors as quantified by microdialysis, increases the efficiency of chemotherapy and increases the extracellular volume [20], [21]. An increase in extracellular volume greatly increases hydraulic conductivity through tissues, in fact hydraulic conductivity increases exponentially to a volume increase [22]. The present finding that treatment with STI571 increases the extracellular volume in KAT-4 carcinoma is thus likely to in part explain the additive effect of STI571 to chemotherapy; transport of the chemotherapeutic agents would be facilitated when the extracellular volume increases. Indeed, in previous studies we have shown that transport of low molecular weight tracer through experimental PROb colorectal rat carcinoma quantified by microdialysis is increased after treatment with STI571 [6]. This is also indicated by our data showing that treatments resembling the protocols resulting in an increased extracellular volume, *i.e.* STI571 for 4 days, or show a trend to such an increased extracellular volume, *i.e.* PTK/ZK for 4 days together with STI571 for 4 days, gave the greatest effects with regard to promoting the anti-tumor effects of Taxol. The failure of the intermittent combination therapy to further potentiate the effect of Taxol could be due to that the decreased vessel leakiness (Fig. 5) prevented an increase in the ECV, thereby inhibiting fluid convection [23]. Further studies are needed to determine the optimal timing of the delivery of the various inhibitors to lower IFP and increase the fluid convection in order to potentiate chemotherapy. Our data suggest that combination treatment with anti-PDGFR and anti-VEGFR tyrosine kinase inhibitors can be used to efficiently lower the IFP of KAT-4 tumors, but that the timing appears to be crucial. Whereas the monotherapies improved the therapeutic effect of Taxol, no additional benefit of the combination therapy was observed.

## Methods

### Cell Culture

The human carcinoma KAT-4 (American Type Culture Collection) was used in this study. KAT-4 cells were originally described as thyroid cancer cells [24], but have recently been shown to resemble the human colorectal adenocarcinoma cell line HT-29 and a thyroid origin of the KAT-4 carcinoma has been questioned [25]. Cells were cultured in RPMI 1640 medium (Sigma) supplemented with 10% fetal bovine serum and antibiotics (100 U/ml penicillin and 100 µg/ml streptomycin). Cultured KAT-4 cells produce and release a non-heparin binding isoform of VEGF-A [10].

### Antibodies

Goat anti-mouse CD31/PECAM-1 antibody was obtained from SantaCruz Biotechnology (Santa Cruz, CA), monoclonal antibodies against Ki67 (TEC3) and desmin were from DAKO (Glostrup, Denmark), and a rabbit polyconal antiserum against cleaved caspase-3 from Cell Signaling Technology (Denvers, MA).

### Tumor Growth and Treatment

Animal experiments were approved by the local ethics committees at the universities in Bergen and Uppsala, and performed according to the UKCCCR guidelines [26]. All manipulations were performed under isoflurane (Abbott Scandinavia, Solna, Sweden) gas anesthesia. Six to eight week-old Fox Chase SCID mice (M&B, Ry, Denmark) received subcutaneous inoculation in the dorsal skinfold of 2×10^6^ KAT-4 cells suspended in 100 µL of PBS. Tumor length and width were measured using calipers, and tumor volume was calculated as π/6×length×width×width. When the tumor volume reached 400 mm^3^ for IFP measurements, mice were randomized to receive p.o. gavage of either vehicle (1% Tween 80, 5% DMSO), 100 mg/kg/day STI571 for 4 days, 25 mg/kg/day PTK/ZK either for 2 days or for 4 days, or 100 mg/kg/day STI571 together with 25 mg/kg/day PTK/ZK for 4 days (termed long term combination treatment) or 100 mg/kg/day STI571 for 4 days followed with 25 mg/kg/day PTK/ZK for last 2 days (termed short term combination treatment). Inhibitors were provided by Novartis Pharma AG, Basel, Switzerland. In the Taxol study, when the tumor volume reached 100 mm^3^ mice were given vehicle, STI571 or PTK/ZK alone for 21 days. Additionally, STI571 was combined with PTK/ZK daily (named continuous treatment) or STI571 was given for 4 days in combination with PTK/ZK for the last 2 days prior to Taxol injection (named intermittent treatment). Taxol (Bristol-Myers Squibb AB, Sweden) at 5 mg/kg was given on day 4, 11 and 18 of the treatment schedule. Animals were euthanized by intraperitoneal injection of 90 mg/kg pentobarbitone. Tumors were subsequently removed surgically, weighed and fixed in 4% paraformaldehyde overnight.

### Measurement of Tumor IFP

Tumor IFP was measured by the wick-in-needle technique, as described previously [6], [20]. Briefly, a standard 23-gauge needle filled with nylon floss and saline, supplemented with 50 IE/mL of heparin, was inserted into the centre of the tumor and connected to a pressure transducer for a period of 30 min. This setup enables stable and continuous recording of fluid pressure. The catheter was clamped during the measurement to verify that the pressure returned to the initial value with a deviation of no more than 1 mmHg. Tumor IFP was measured earliest 4 h after the last administration of vehicle, STI571, PTK/ZK or combinations of both drugs. IFP was measured at two separate locations in the tumor and the mean was calculated for each tumor. There was no difference in the growth rate among the groups at the day of measurement (data not shown).

### Immunohistochemistry and Stereological Analyses of Tumor Vessels

Excised tumors were embedded in paraffin, and 4 µm thick sections were cut onto Superfrost Plus slides (Histolab, Gothenburg, Sweden). The sections were deparaffinized and boiled in 10 mM citrate buffer, pH 6.0 for 2×7 min at 750 W in a microwave oven. Tissue peroxidase activity was quenched by incubation with 3% H_2_O_2_ in PBS for 10 min, and unspecific antibody binding was blocked by incubation with 20% serum species-matched to the secondary antibody. Endothelial cells were stained with a CD31/PECAM-1 antibody (4 µg/mL), and a desmin antibody was used to stain pericytes (1∶50). Antibodies against cleaved caspase-3 (1∶200) and Ki-67 (1∶25) were used to stain for apoptotic and proliferative cells, respectively. The fraction of cleaved caspase-3 positive cells or Ki67 positive cells was determined after analyzing 1,000 cells. For pericyte quantification, the number of pericyte nuclei associated with tumor vessels was counted. Omission of primary antibody was used as a negative control. Positive reactions were developed using DAB (Vector Laboratories, Inc., Burlingame, CA) as a peroxidase substrate. Sections were counterstained in Mayer's hematoxylin, dehydrated, and coverslipped in Mountex resin (Histolab, Gothenburg, Sweden). Stereological quantification of capillary tumor blood vessels was performed after CD31 staining. Vision fields were selected by screening tumor sections every 1 mm. Images of the vision fields (0.09 mm^2^) were captured, blood vessels and viable area were manually marked and vessel area and vessel perimeter was quantified using digital image processing and analysis software “Leica QWin Standard” in 9–40 vision fields (depending on the size of the sections) of one section from each animal.

### Determination of Extracellular and Plasma Volumes

Extracellular volume (ECV) and plasma volume (PV) in tumor and skin were determined by the dilution principle using radioactive-labelled isotopes [10]. Total tissue water (TTW) was determined as (wet weight-dry weight)/dry weight. Tissue samples were dried at 50°C until constant weight (normally 3–4 weeks). The distribution volumes for ^51^Cr-EDTA (Institute of Energy Technology, Kjeller, Norway) and ^125^I-labeled human serum albumin (^125^I-HAS); Institute of Energy Technology, Kjeller, Norway), measuring ECV and PV respectively, were calculated as plasma equivalent volumes, *i.e.* counts per min per mg of tissue divided by counts per min per ml of plasma. Both isotopes were given after functional nephrectomy by the bilateral ligation of the renal pedicles via flank incision. ^51^Cr-EDTA (300,000 cpm in 0.2 mL PBS) was injected into the catheter in the tail vein and left to circulate for 85 min before injecting ^125^I-HAS (3×10^6^ cpm in 0.2 mL PBS). Blood samples were taken 5 min later, by heart puncture. Skin from the back was used as a reference control. Radioactivity was determined in a COBRA II, Auto-gamma counter (Packard) with automatic background and spillover correction.

### Vascular Leakage Assay

Evans blue (30 mg/kg) was administered via tail vein injection 30 minutes before sacrifice. Tumors were excised, dried at 60°C over-night and weighed, followed by extraction in 1 mL formamide at 55°C over night, and the content was quantified by reading at 600 nm in a spectrophotometer [27]. Additionally, blood samples were taken and the amount of Evans blue in the tumor was related to the amount of the Evans blue in the serum.

### Statistical Methods

Statistical analysis of the IFP measurements and tumor growth assays were analyzed with One-way analysis of variance, with a subsequent post hoc analysis with Duncan's adjustment. The experiments determining the total tissue water, extracellular volume and plasma volume were analyzed with Kruskall-Wallis One Way Analysis on Ranks when the normality test failed with a subsequent post hoc analysis using Dunn's test. P<0.05 was considered statistically significant.

## Supporting Information

Figure S1 Effects of PDGF and VEGF receptor kinase inhibitors on vessel morphology and pericyte coverage. Four Âµm thick sections of KAT-4 tumors from mice treated with vehicle, STI571 or PTK/ZK, alone or in combination, were stained for endothelial cells of blood vessels by CD31 antibodies and for pericytes by desmin antibodies. Endothelial cells are stained dark blue with alkaline phosphatase and the pericytes are stained brown using DAB as peroxidase substrate. Tumor cells are counterstained with hematoxylin. The bars represent 20 Âµm. (A) vehicle, (B) STI571 4 days, (C) PTK/ZK 2d, (D) PTK/ZK 4d, (E) STI571 4d and PTK/ZK 2d, (F) STI571 4d and PTK/ZK 4d. Arrows point at desmin positive pericytes and arrowheads at CD31 positive endothelial cells. The areas within red squares were enlarged and put on top of the original photos.
(3.06 MB EPS)

Figure S2 Effect of PDGF and VEGF receptor kinase inhibitors on KAT 4 tumor cell proliferation. Sections of KAT 4 tumors from mice treated with vehicle, STI571 or PTK/ZK, alone or in combinations, were stained for Ki67 to monitor tumor cell proliferation. Ki67 positive cells are stained brown using DAB as peroxidase substrate. Tumor cells are counterstained with hematoxylin. The bars represent 20 Âµm. (A) vehicle, (B) STI571 4d, (C) PTK/ZK 2d, (D) PTK/ZK 4d, (E) STI571 4d and PTK/ZK 2d, (F) STI571 4d and PTK/ZK 4d. Arrows point at Ki67 positive cells.
(2.17 MB TIF)

Figure S3 Effect of PDGF and VEGF receptor kinase inhibitors on KAT 4 tumor cell apoptosis. Sections of KAT 4 tumors from mice treated with vehicle, STI571 or PTK/ZK, alone or in combinations, were stained for cleaved caspase-3 to monitor tumor cell apoptosis. Cleaved caspase-3 positive cells are stained brown using DAB as peroxidase substrate. Tumor cells are counterstained with hematoxylin. The bars represent 20 µm. (A) vehicle, (B) STI571 4d, (C) PTK/ZK 2d, (D) PTK/ZK 4d, (E) STI571 4d and PTK/ZK 2d, (F) STI571 4d and PTK/ZK 4d. Arrows point at cleaved caspase-3 positive cells.
(2.17 MB TIF)
