# Supplementary figures and images for: Combined Anti-Angiogenic Therapy Targeting PDGF and VEGF Receptors Lowers the Interstitial Fluid Pressure in a Murine Experimental Carcinoma
Source: PLoS One. 2009 Dec 4;4(12):e8149. doi: 10.1371/journal.pone.0008149 (PMC2781164; doi:10.1371/journal.pone.0008149)

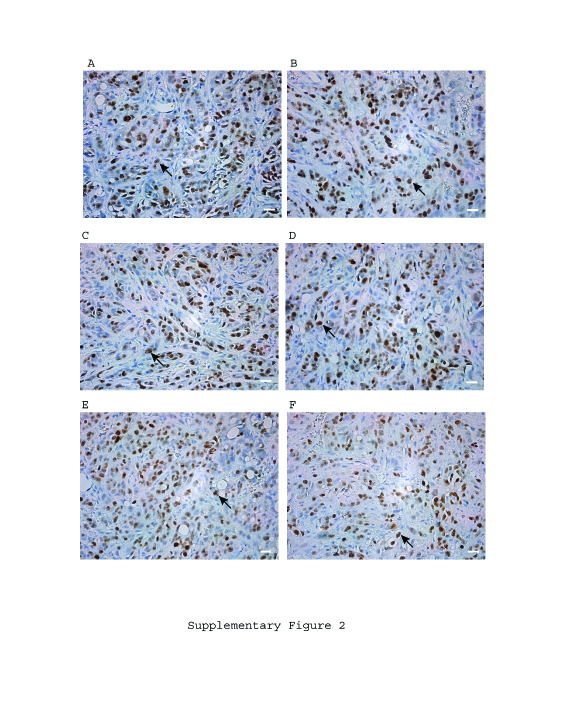

Supplement: Figure S2 — Effect of PDGF and VEGF receptor kinase inhibitors on KAT 4 tumor cell proliferation. Sections of KAT 4 tumors from mice treated with vehicle, STI571 or PTK/ZK, alone or in combinations, were stained for Ki67 to monitor tumor cell proliferation. Ki67 positive cells are stained brown using DAB as peroxidase substrate. Tumor cells are counterstained with hematoxylin. The bars represent 20 Âµm. (A) vehicle, (B) STI571 4d, (C) PTK/ZK 2d, (D) PTK/ZK 4d, (E) STI571 4d and PTK/ZK 2d, (F) STI571 4d and PTK/ZK 4d. Arrows point at Ki67 positive cells. (2.17 MB TIF) [file pone.0008149.s002.tif]

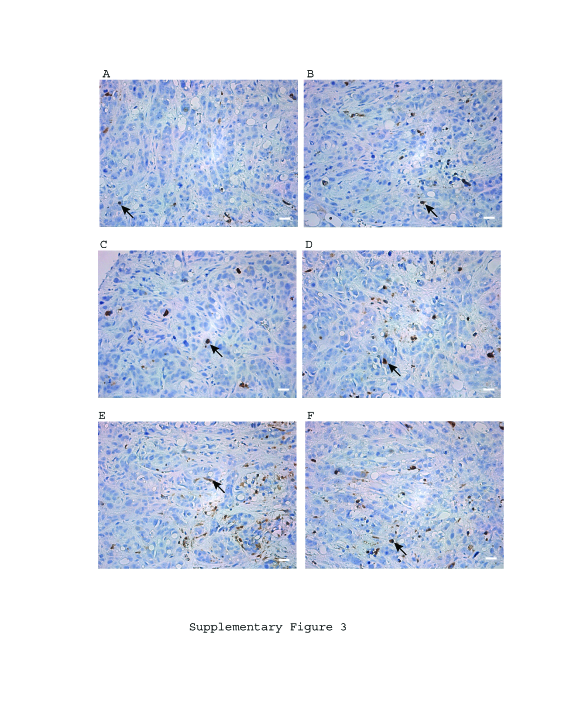

Supplement: Figure S3 — Effect of PDGF and VEGF receptor kinase inhibitors on KAT 4 tumor cell apoptosis. Sections of KAT 4 tumors from mice treated with vehicle, STI571 or PTK/ZK, alone or in combinations, were stained for cleaved caspase-3 to monitor tumor cell apoptosis. Cleaved caspase-3 positive cells are stained brown using DAB as peroxidase substrate. Tumor cells are counterstained with hematoxylin. The bars represent 20 µm. (A) vehicle, (B) STI571 4d, (C) PTK/ZK 2d, (D) PTK/ZK 4d, (E) STI571 4d and PTK/ZK 2d, (F) STI571 4d and PTK/ZK 4d. Arrows point at cleaved caspase-3 positive cells. (2.17 MB TIF) [file pone.0008149.s003.tif]
